# The Ins and Outs of Messenger RNA Electroporation for Physical Gene Delivery in Immune Cell-Based Therapy

**DOI:** 10.3390/pharmaceutics13030396

**Published:** 2021-03-16

**Authors:** Diana Campillo-Davo, Maxime De Laere, Gils Roex, Maarten Versteven, Donovan Flumens, Zwi N. Berneman, Viggo F. I. Van Tendeloo, Sébastien Anguille, Eva Lion

**Affiliations:** 1 Tumor Immunology Group, Laboratory of Experimental Hematology, Faculty of Medicine and Health Sciences, Vaccine & Infectious Disease Institute (VAXINFECTIO), University of Antwerp, 2610 Wilrijk, Belgium; Gils.Roex@uantwerpen.be (G.R.); maarten.versteven@uantwerpen.be (M.V.); donovan.Flumens@uza.be (D.F.); zwi.berneman@uza.be (Z.N.B.); viggovantendeloo@gmail.com (V.F.I.V.T.); sebastien.anguille@uantwerpen.be (S.A.); 2 Center for Cell Therapy & Regenerative Medicine, Antwerp University Hospital, 2650 Edegem, Belgium; maxime.DeLaere@uza.be; 3 Division of Hematology, Antwerp University Hospital, 2650 Edegem, Belgium

**Keywords:** messenger RNA, gene delivery, electroporation, in vitro transcription, immune cell-based therapy

## Abstract

Messenger RNA (mRNA) electroporation is a powerful tool for transient genetic modification of cells. This non-viral method of genetic engineering has been widely used in immunotherapy. Electroporation allows fine-tuning of transfection protocols for each cell type as well as introduction of multiple protein-coding mRNAs at once. As a pioneering group in mRNA electroporation, in this review, we provide an expert overview of the ins and outs of mRNA electroporation, discussing the different parameters involved in mRNA electroporation as well as the production of research-grade and production and application of clinical-grade mRNA for gene transfer in the context of cell-based immunotherapies.

## 1. Introduction

**Figure 1:**
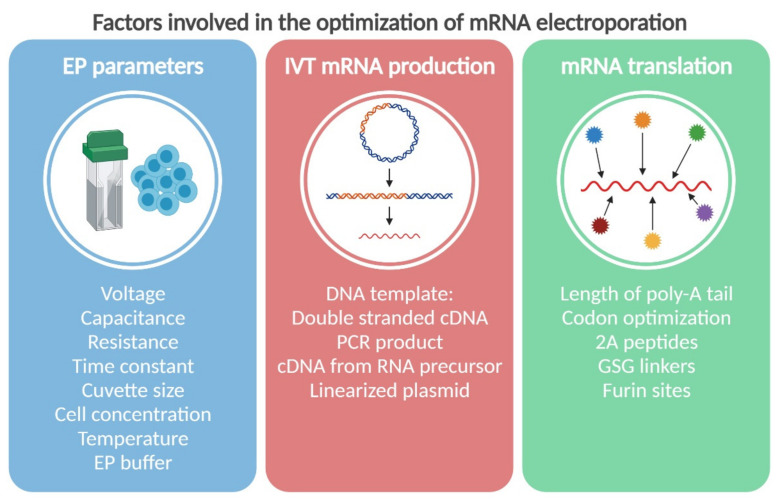
Overview of the main factors that influence the success of a messenger (mRNA) electroporation-based therapy. Several factors may influence the transfection efficiency (blue), synthesis (red) and translation (green) of mRNA in electroporation-based therapies. These factors can be individually optimized, combined and tailored for each type of immune cell and target gene to be transferred. EP, electroporation; IVT, in vitro transcription; cDNA, complementary DNA; PCR, polymerase chain reaction. Created with BioRender.com.

Since the early experimental application of electric pulses in the field of medicine during the eighteenth century, electroporation has become a universal method for transfecting biological and synthetic compounds into an array of prokaryotic and eukaryotic cells for a wide number of purposes [1]. Electroporation, also called electropermeabilization, is defined as the application of voltage pulses that generate an electric field between two electrodes that disrupts the integrity of a cell membrane, allowing the formation of pores. It was first developed as an irreversible process of pore formation that did not allow recovery of the cell membrane, therefore resulting in cell death [1] Reversible electroporation was introduced in 1957 by Stämpfli and Willi [2], but it was not until 1982 that this type of electroporation was described for the transfection of genetic material [3]. In that article, Neumann et al., who also coined the term “electroporation”, described how electric pulses enhanced the uptake of extracellular DNA into mouse cells [3]. Since then, the versatility of this technique has been demonstrated in multiple cell types and organisms for the transfection of various molecules in a wide range of applications. The field of cell-based immunotherapy in particular has made enormous progress due to the development and optimization of messenger RNA (mRNA) electroporation for gene transfer. This type of genetic engineering, compared to that of the viral delivery of genes, represents a safer alternative for protein expression with no risk of insertional mutagenesis and lower immunogenicity [4,5]. The superiority of mRNA electroporation over passive pulsing or lipofection of mRNA, and even over plasmid DNA electroporation, in terms of efficiency of gene delivery was demonstrated by our group two decades ago [6,7]. In contrast to plasmid DNA electroporation, transient gene expression linked to the natural decay of introduced mRNA provides an accurate system to control the synthesis of exogenous proteins. The main factors involved in successful mRNA electroporation for gene transfer can be divided into three main categories, (i) electroporation parameters, (ii) variables of in vitro mRNA synthesis, and (iii) elements used to enhance transfected mRNA stability and transgene expression (Figure 1). In this review, we discuss the different parameters marking mRNA electroporation and how to implement them as well as the factors involved in the production of clinical-grade mRNA for electroporation in the context of cell-based immunotherapies.

## 2. The Physics: Parameters of Electroporation

**Figure 2:**
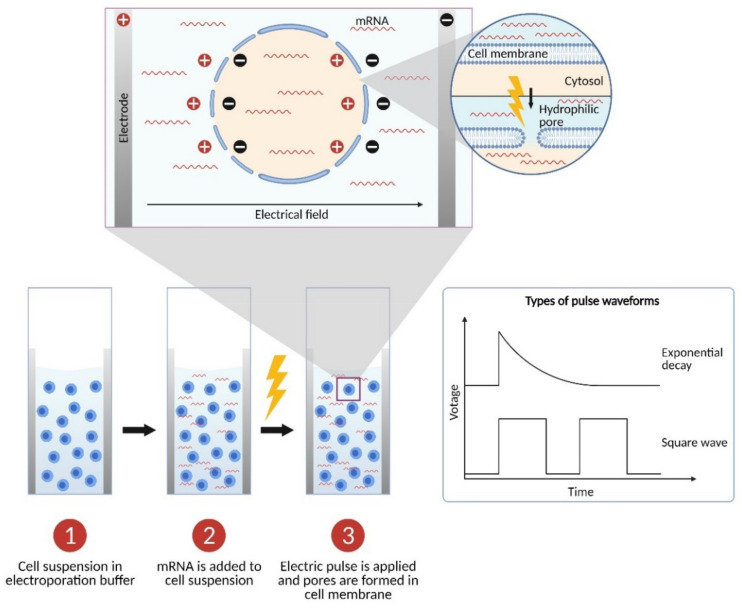
Elements of the electroporation process. The electroporation cuvettes contain two parallel electrodes separated by a gap where the cell suspension is placed. The cells that are in suspension in an electroporation buffer (**1**) are mixed with mRNA (**2**) and pulsed (**3**) with one of the two main types of electric waves: the exponential decay or the square wave. During the electric pulse, pores are transiently formed in the cell membrane through which the mRNA can flow into the cytosol. Created with BioRender.com.

Electrical disruption of a cell membrane causes the formation of pores through which nucleic acids, proteins, and other small molecules present in the environment surrounding the cells can permeate, gaining access to the intracellular space (Figure 2). In vitro electroporation of immune cells is carried out using a pulse generator (or electroporator). Generally, cells are placed in sterile cuvettes consisting of a cell chamber with two parallel metal electrode plates. Commercially available cuvettes for the transfection of mammalian cells typically have a gap size of 2 or 4 mm. The difference in electric potential between the two electrodes is called voltage (*V*) and it is measured in volts (V). Before electroporation, cell membranes are in a non-permeable state that is characterized by low conductivity, dielectrical constant, and polarizability [8]. As mentioned in the previous section, a voltage pulse is applied during the electroporation process. This generates an electric field that creates a linear strength gradient between the electrodes. The voltage used divided by the gap size of the cuvette determines the electric field strength (*E*), commonly expressed in kilovolts per centimeter (kV/cm). That electric field will create an induced cell membrane potential. If the field strength is high enough, the induced cell membrane potential will surpass a threshold potential in which the cell membrane will undergo polarization and dielectric breakdown followed by an increase in membrane conductivity and permeability [9]. These changes allow the creation of hydrophilic nanopores through which ions in aqueous solutions may pass [10] (Figure 2). That flow of extracellular components will lead to the formation of larger pores allowing the passage of larger molecules such as RNA [10]. Moreover, ions moving from the positive electrode to the negative electrode will create electrophoretic forces that allow RNA (a polyanion) to travel to the positive electrode [8]. The induced cell membrane potential is not uniform nor permeabilization occurs homogenously across the membrane [8,11]. In fact, more pores will be created at the side of the cell membrane that faces the negative electrode, whereas, at the side of the positive electrode, a larger area of the cell membrane will be permeabilized (Figure 2). When the electric field is removed at the end of the electroporation process, resealing of the cell membrane occurs. Contrary to pore formation, which takes place within milliseconds, resealing of the cell membrane may take from minutes to hours [8]. When a critical field strength is reached, resealing of the cell membrane may not be possible, leading to cell death.

The electroporation process is also influenced by other parameters, including the capacitance, resistance, time constant, and pulse duration [12]. Capacitance (*C*), measured in microfarads (µF), is defined as the ability of a capacitor—in this case, the membrane of the cells in suspension—to retain a charge (*Q*) in the form of a potential difference or voltage. Accordingly, capacitance follows the equation: *C = Q/V*. Resistance (*R*), expressed in ohms (Ω), is the force against the electrical current, which is influenced by elements such as the cell suspension or electroporation buffer. Taking into account these parameters, two types of pulses (or waveforms) are commonly used for electroporation of immune cells, exponential decay and square waves (Figure 2). Exponential decay is a pulse in which the chosen voltage is reached at the beginning of the pulse followed by an exponential and rapid decrease to zero [12]. That decay follows the formula:

(1) $$V_{t}=V_{0}\left(e^{-\left(t/\tau \right)}\right),$$

where *V*_0_ is the initial voltage at which the capacitor is charged, *V_t_* is the voltage at a time *t*, and *τ* is the time constant at which the voltage of the pulse has decreased from *V*_0_ to *V*_0_*/e* [12]. The time constant results from the combination of the resistance and capacitance (*τ = R × C*). The time constant should not be confused with pulse length or duration of the pulse (*τ*). An alternative form of exponential decay pulse—usually called “time constant”—applies a voltage for a certain amount of time without constraining the capacitance. When the time is kept constant, the capacitance is adjusted to reach a particular (constant) pulse length for all the test conditions, which is dependent on the resistance of the cell suspension and the volume in the cuvette. In contrast, the square wave pulse, which gets its name from the shape of its electric potential curve, maintains the same voltage for the entire duration of the pulse after which it returns to a voltage of zero [12]. With this type of wave, it is possible to apply multiple and repeated electric pulses during a single electroporation.

The electric field strength, together with the duration of the pulse, is key in maintaining cell viability and transfection efficiency during electroporation (reviewed by [13]). Apart from its implications regarding the field strength, gap size will also determine the electroporation buffer volume and number of cells a cuvette can contain. Thus, widening the gap size will increase the usable volume and number of cells, but it will also reduce the field strength. Other parameters affecting the success of electroporation include the electroporation buffer used, the temperature of the different components during electroporation, and the cell concentration. The conductivity of the electroporation buffer, marked by its salt content, and the cell concentration are two of the main parameters that affect the resistance of the sample during electroporation [14]. Moreover, the composition of the buffer, especially the content in salts and sugars, may have a negative effect on cell viability and transfection efficiency [15,16,17]. Related to this, the presence of remaining salts in the cell and nucleic acid suspension may increase the final concentration of salts in the electroporation mixture leading to arcing [18]. Arcing is a complete or partial discharge of an electric current in a sample easily recognizable as an audible popping sound. This phenomenon occurs in the presence of high salt concentrations, but also in the presence of bubbles, of oil on the electrodes of the cuvettes due to handling without gloves, or with faulty cuvettes in combination with high voltages, and negatively impacts cell viability. The temperature of the electroporation buffer, cuvette, and cells is another variable to be considered during the optimization of electroporation conditions [12]. For example, keeping the cell suspension on ice or at 4 °C may limit membrane plasticity, reducing electroporation efficiency; however, cell viability and yield are often improved at lower temperatures. With respect to the recovery medium after permeabilization, there is no clear rule; however, a general recommendation to improve cell viability and pore resealing, which occurs within seconds, is the addition of human or non-human serum, depending on the experimental requirements [19]. Taken together, each of these parameters and elements of electroporation can be optimized to improve the efficiency of mRNA delivery while maintaining cell viability and yield [12,20,21].

## 3. The Chemistry: In Vitro Synthesis of mRNA for Electroporation

For mRNA electroporation in gene transfer studies, one of the key factors at the molecular level for efficient and correct protein expression is the synthesis of the mRNA. In eukaryotic cells, the first step of gene expression occurs in the nucleus and consists of the transcription of an mRNA strand from a segment of complementary DNA (cDNA) by RNA polymerase II. Before being transported to the cytoplasm to be translated into protein, the precursor mRNAs (pre-mRNAs) undergo a maturation process in the nucleus that includes modification of the 5′ and 3′ ends and elimination of the non-coding regions (introns). The first modification occurs at the 5′ region where a methylated guanosine or “cap” is added to the first nucleotide of the pre-mRNA, protecting it from degradation by exonucleases [22]. Next, polyadenylation takes place at the 3′ terminus of the pre-mRNA [23]. Finally, the introns are removed from the pre-mRNA through splicing, leaving a mature mRNA consisting of the protein-coding regions (exons) flanked by untranslated regions (UTRs), the methylated guanosine cap, and a poly(A) tail.

The 3′ UTR region of the mRNA is a primary factor influencing its cellular localization, stability, and translation efficiency [24]. Messenger RNAs encoding the same protein can exhibit different 3′ UTR isoforms depending on the specific intended fate of a particular mRNA. Importantly, the length and composition of the 3′ UTR region help regulate the mRNA, and thereby the protein levels in a cell at any given time. In fact, the 3′ UTR region, together with the 5′ cap, is indispensable for the formation of the stem-loop structure needed to initiate mRNA translation. Shorter 3′ UTRs have an advantage in the formation of the initiation loop compared to that of mRNAs with longer 3′ UTRs. In the nucleus, polyadenylation of mRNAs on their 3′ side is a tightly regulated and standardized process that results in the addition of approximately 200 nucleotides in mammals [25]. The length of the poly(A) tail is usually shortened after the mRNA enters the cytoplasm through a mechanism that is involved in regulating mRNA decay [26]. Actually, the poly(A) tail is a dynamic region of the mRNA sequence that is affected by the processes of adenylation (to lengthen) and deadenylation (to shorten), which are adjusted during different stages of the cell cycle or in response to specific signals. The effect of poly(A) tail length on translational control has been previously reviewed by Weill et al. [27].

**Figure 3:**
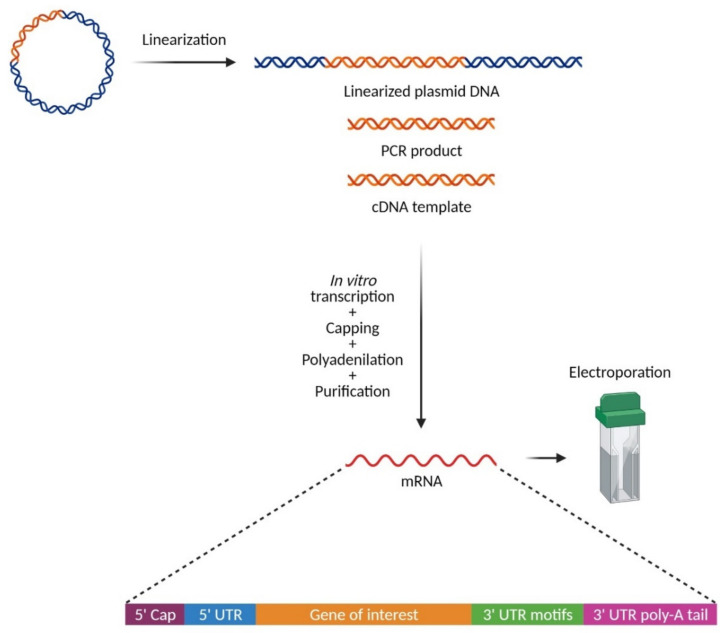
Basic workflow for mRNA synthesis. The in vitro synthesis of mRNA starts with the preparation of the DNA template containing the gene of interest (depicted in orange), which can be linearized plasmid DNA, a PCR product, or a cDNA template. These DNA templates will be used for the in vitro transcription of mRNA using an RNA polymerase, followed by mRNA capping at the 5′ untranslated region, addition of a poly(A) tail at the 3′ untranslated region (optional in cases were a poly(A) is included in the DNA template), and purification of the final mRNA. UTR, untranslated regions. Created with BioRender.com.

Most natural mRNAs are degraded by endonucleases or exonucleases within minutes or hours of being transcribed. However, transcripts that encode proteins which are functionally vital for the cell are usually more stable. An important determinant of mRNA stability lies in the portion of the 3′ UTR preceding the poly(A) tail. In particular, human globin mRNAs have been characterized as being highly stable with half-lives up to 48 h due to their 3′ UTR [28]. Therefore, the addition of these 3′ UTR motifs to synthetic mRNAs benefits their stability, resulting in higher protein expression levels [29]. In situations where increased protein translation is needed without wanting to affect the mRNA half-life, addition of the cytochrome b-245 alpha chain gene 3′ UTR may be a suitable candidate [30]. In the laboratory, mRNA synthesis is commonly performed via in vitro transcription (IVT), a rapid and efficient technique that yields high amounts of mRNA. The open reading frame (ORF) of the therapeutic gene of interest is preceded by a 5′ UTR containing a promoter and the Kozak sequence [31]. The promoter is usually specific for bacteriophage SP6, T3, or T7 RNA polymerase [32,33,34,35]. The Kozak consensus elements, called the Shine–Dalgarno sequence in prokaryotes, are the nucleotides preceding and following the AUG start codon. These sequences at the proper position in vertebrates act as enhancers of initiation of translation [36]. The ORF of the gene of interest is followed by a 3′ UTR and a poly(A) tail, depending on the template used. The 3′ UTR and a poly A tail are elements crucial for the stability and translational efficiency of the produced mRNA. To generate IVT mRNA, there is a broad range of commercially available IVT kits; however, the basic requirements to initiate transcription are a purified cDNA template, ribonucleotide triphosphates, distilled water, reaction buffer, and an RNA polymerase. The double-stranded cDNA template is typically a product of polymerase chain reaction (PCR), cDNA from an RNA precursor, or a linearized plasmid DNA (Figure 3). In the case of PCR products, the gene of interest is amplified by PCR using a plasmid or genomic DNA as template. Then, through the addition of the appropriate primers and another round of PCR amplification, the cDNA template is linked to a promoter for the ultimate translation of the ORF [37]. This is done by including at the 5′ end of one of the primers the promoter region of an RNA polymerase from one of the bacteriophages. When using cDNA generated from an RNA precursor, the RNA first undergoes a reverse transcription reaction with primers containing the bacteriophage polymerase, resulting in the production of a single DNA strand bound to the RNA precursor. The second cDNA strand is then generated using the complementary RNA as primer to form the double-stranded DNA. For plasmids, the circular DNA is linearized by digestion with a restriction enzyme prior to IVT, to prevent the transcription of the entire plasmid sequence. This results in the creation of either blunt end or sticky 3′-overhanging ends, depending on the enzyme used. Related to this, an important concern after plasmid linearization is the addition of non-adenine nucleotides to the poly(A) tail from the overhanging ends, which otherwise will reduce translation efficiency. To avoid non-adenine nucleotides at the end of the poly(A) tail, type IIS restriction enzymes can be used instead of the classical type II enzymes as type IIS enzymes cleave the DNA sequence outside the recognition site and create blunt ends without 3′ overhangs. A detailed protocol has been previously published [38].

## 4. The Biology: How to Improve mRNAs for Better Stability and Translation

Apart from optimizing the electroporation conditions and choosing the best template for mRNA production, other factors also contribute to successful mRNA stability and translation and should be considered to improve protein expression in electroporated cells. As described in the previous section, mRNA capping and polyadenylation are indispensable for successful mRNA translation. The 5′ capping of IVT mRNA can be directly done during RNA generation or done separately. The various options for 5′ capping have been reviewed elsewhere [39]. When polyadenylation is performed separately after IVT, mRNAs are formed with a greater variability in poly(A) tail length. In other cases, the poly(A) tail is cloned into the plasmid and positioned within the construct after the ORF. Since poly(A) tails are shortened in the cytoplasm due to natural mRNA degradation, different plasmids have been developed based on the extension of the poly(A) tails to improve mRNA yield and stability. For example, the pST1-A120 vector includes a poly(A) tail of ~120 base pairs (bp) [29], and the plasmid pEVL can be used to increase the poly(A) tail length up to ~500 bp [40]. Some plasmids for in vitro synthesis of RNA can be purchased from commercial sources, such as pGEM-XZ and pSPXX vector series (Promega), pBluescript II phagemid vectors (Agilent), pCRII and pTRIPLEscript vectors (Invitrogen) [41], pT7-mRNA vector (VectorBuilder Inc.), and pMRNAxp mRNAExpress vector (System Biosciences). Another factor that improves mRNA translation is codon optimization. Some mRNAs may contain “rare” codons that decrease the rate of translation, an issue that has been previously reviewed [42]. Codon optimization involves replacing those codons with more highly expressed synonymous codons, thereby enhancing protein expression compared to that of the native sequences [43].

Gene transfer using mRNAs may encode for multiple proteins at the same time, similar to what can be done using DNA vectors. Over the years, various strategies have been used in gene therapy to yield individual translation products from polycistronic constructs [44]. Two of the most common strategies are the insertion of internal ribosome entry sites (IRES) and self-cleaving 2A peptides sequences between the genes. IRES were first discovered in picornavirus and allow cap-independent translation of proteins (reviewed by [45]). Placed between two independent sequences, IRES are able to recruit ribosomes to initiate the translation of the downstream genes [45]. However, due to the large size and inconsistent translation rates of IRES, this system has become less popular in mRNA gene transfer in favor of 2A peptides [46,47]. Initially found in picornavirus, 2A peptides are 18–22 amino acid-long oligopeptides that are part of the ribosome “skipping” translational mechanism [46]. They allow for the stoichiometric expression of upstream and downstream genes in bicistronic cassettes and exhibit a high cleaving efficiency with minimal addition of amino acids to the translated proteins. Among the various 2A peptides, P2A from porcine teschovirus-1 and T2A from *Thosea asigna* virus usually yield better results in comparative studies than that of other 2A peptides, such as F2A from foot-and-mouth disease virus or E2A from equine rhinitis A virus [46]. Multiple 2A peptides can also be used together in multicistronic constructs, resulting in different gene expression levels depending on the combination of peptides used [48]. An important factor that may limit cleavage efficiency is the C-terminal sequence preceding the 2A peptide [49,50]. Frequently, 2A peptides are preceded by flexible oligopeptide linkers that are comprised of combinations of glycine and serine, in many cases being the combination Gly-Ser-Gly [50,51]. These spacers improve the cleaving efficiency of the 2A peptides, resulting in the correct expression of the upstream and downstream proteins [50,51,52]. However, they also add a few more amino acids to the C-terminus of the upstream protein, potentially having functional consequences that must be assessed on a case-by-case situation. A solution to this problem is the addition of furin recognition sites before the 2A peptide [52,53]. Furin is an endoprotease that recognizes RX(K/R)R motifs. The 2A peptides, glycine-serine linkers, and furin cleavage sites can be used simultaneously [51,52]. However, it is important to note that they must be in a single ORF with the genes of interest either before and/or after them. This ensures the correct translation and expression of the transferred proteins.

## 5. Clinical Production of mRNA for Electroporation

In general, two types of clinical-grade mRNA can be distinguished: documented-grade [54] and good manufacturing practice (GMP)-grade mRNA. These two categories of mRNA vary in the regulatory aspects associated with their production, which are determined by the intended usage of the mRNA (i.e., as a starting material or as a medicinal product), the class of advanced therapy medicinal product (ATMP) the final product belongs to (i.e., cell-based ATMP or gene therapy product), and the stage of development of the medicinal product (i.e., investigational or marketed). In the context of mRNA transfection for immune cell-based immunotherapeutics, mRNA can be considered both starting material and active substance for the generation of a cell-based ATMP. While Directive 2001/83/EC [55], as amended, holds the obligation for the manufacturing authorization holders to use only active substances that have been manufactured in accordance with GMP for starting materials, Directive 2005/28/EC includes no such requirement for manufacturers of investigational medicinal products [56]. For this reason, mRNA not fully complying with the GMP requirements, but of which the quality is controlled and documented in such a way that it justifies its use in the clinical setting (i.e., documented-grade mRNA) is a valid starting material for the production of mRNA-modified cell-based investigational medicinal products. For any other clinical application, GMP-grade mRNA is required, according to the applicable regulatory guidelines. Guidance on the interpretation of the GMP principles and guidelines for active substances used as starting materials are described in “The Rules Governing Medicinal Products in the European Union“ (EudraLex), Volume 4 “Good Manufacturing Practice”, Part II “Basic Requirements for Active Substances used as Starting Materials” as laid down in Directive 2003/94/EC [57]. 

**Figure 4:**
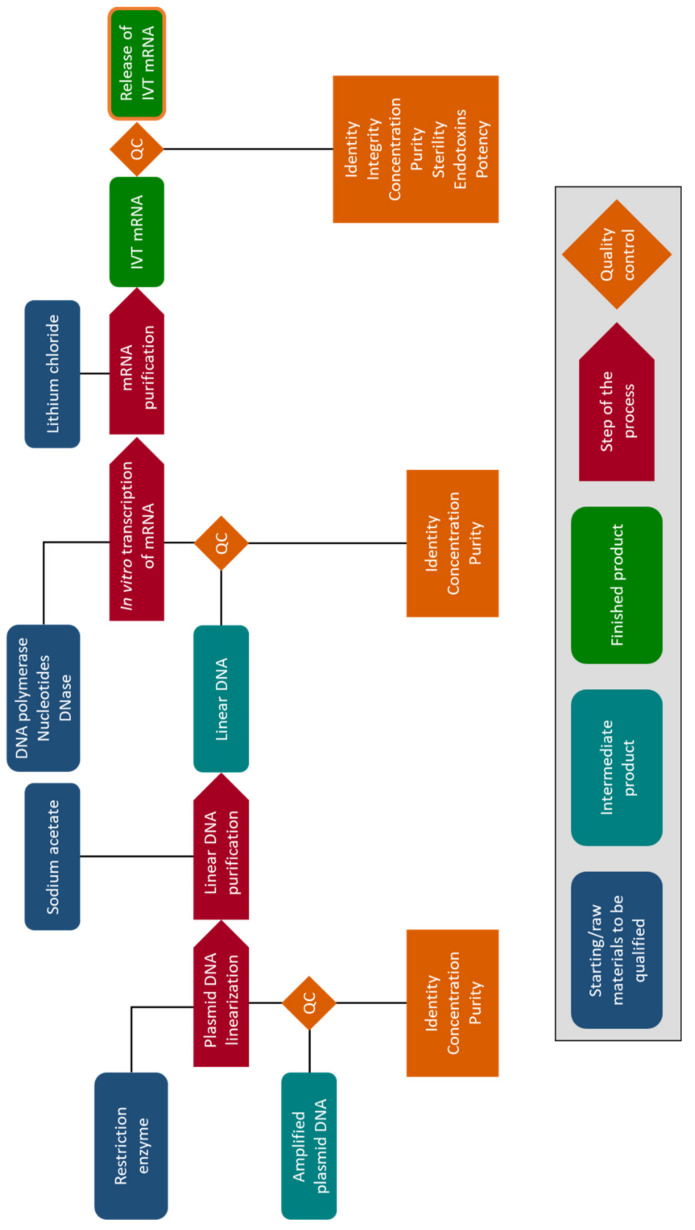
Example of the messenger RNA production processes and quality control testing for the release of IVT mRNA in a clinical setting for human use. Generally, different reagents, raw materials, and intermediate products are needed to produce any in vitro transcribed (IVT) mRNA. However, compared to research-grade mRNA, manufacture, and final release of IVT mRNA for clinical use in humans usually requires more quality controls (QC). These controls include the quantification of the mRNA concentration, purity, and integrity, but also the confirmation of the identity the mRNA, its sterility, its potency, and the absence of potentially damaging endotoxins.

For the production of GMP-grade mRNA, an extensive documented quality management system needs to be established. This system should cover the complete process of active pharmaceutical ingredient (API) manufacturing, from qualification of raw material suppliers, overproduction, quality control, release of intermediates and the API, to API packaging, labeling, storage, and distribution. The EudraLex GMP guidelines in addition set standards for manufacturing premises, process equipment, and personnel, while also covering administrative aspects such as record keeping, change and deviation management, and corrective action and preventive action (CAPA) system. To ensure the highest quality of the produced mRNA, each batch is subjected to extensive QC testing, which commonly includes assays for integrity, identity, potency, and, as appropriate, sterility and the presence of bacterial endotoxins (Figure 4). QC tests related to detection of relevant impurities, such as residual solvents, proteins, template and/or bacterial DNA, and other mRNA properties (e.g., capping efficiency) depend on the manufacturing process selected and the desired/required degree of control. These procedures should be validated, taking into consideration the relevant guidance and recommendations found in the International Council for Harmonization of Technical Requirements for Pharmaceuticals for Human Use (ICH) Q2 (R1) guidelines (CPMP/ICH/381/95) [58]. The EudraLex GMP guide also includes recommendations (with no obligatory force) for starting materials used in the production of investigational medicinal products. While it is recognized that not all GMP standards are applicable in early clinical development and a certain level of flexibility is required in this phase, manufacturers should still ensure that appropriate GMP concepts are applied in the production of APIs for use in clinical trials and that compliance increases with the stage of development.

From the above, it is evident that producing clinical-grade mRNA requires dedicated infrastructure, equipment, and expertise. Hence, many investigators outsource this activity and purchase customized clinical-grade IVT mRNA from specialized commercial suppliers. Currently, different companies provide these services, which include BioNTech, Biomay CureVac, EtheRNA, and Eurogentec in Europe, and Aldevron, Creative Biolabs, Moderna, and TriLink in the United States of America. Our research group has extensive experience in different clinical trials involving the use of mRNA as API starting material (ClinicalTrials.gov reference number NCT00834002, NCT00965224, NCT01291420, NCT01686334, NCT02649582, NCT02649829). From these clinical studies, we have learned that the service of customized clinical-grade mRNA production is associated with very high costs and extended turn-around-times for production and delivery. This is at least in part due to the fact that, while the amounts of mRNA as API required in the context of early phase clinical trials are relatively small, substantially higher amounts of mRNA need to be produced, at cost, to comply with GMP quality control and stability testing requirements. In this perspective, in-house production of small to medium batches of documented-grade mRNA, which is less demanding in terms of required infrastructure and overall GMP compliance, may provide clinical research centers with an alternative to support their early clinical development needs. It has to be taken into account, however, that any change to the API at a later stage of development made in view of meeting the increasing regulatory requirements, results in the need for comparability studies to ensure these changes do not alter the final cell therapy product. Still, the significantly reduced cost associated with in-house production of documented-grade mRNA versus custom-produced GMP-grade mRNA may ensure sustainability of research efforts focusing on mRNA-electroporated cell-based immunotherapeutics.

## 6. Clinical Application of mRNA Electroporation in Cell-Based Immunotherapies

Electroporation of mRNA as a pharmaceutical tool for transient expression of proteins of interest has been applied as a therapeutic strategy in malignant, infectious, and autoimmune diseases. Loading antigen-presenting dendritic cells (DCs) with tumor-associated antigens (TAAs) alone or in combination with immune-modulating molecules, such as agonists of T-cell activation, is the most common usage of mRNA electroporation in a clinical setting (Table 1). This therapeutic modality focuses on promoting multi-epitope antigen-specific T-cell responses to target tumor cells. Taking this idea further, multiple mRNA encoding different TAAs can be co-electroporated in order to improve immune responses and to avoid immune evasion. Another application, as a safer and more versatile alternative than viral transduction, is the redirection of T cells with immune receptors such as T-cell receptors (TCRs) and chimeric antigen receptors (CARs) to specifically and directly target TAAs presented by tumor cells (Table 2). Although less popular compared to T cells in a clinical context, peripheral blood mononuclear cells and natural killer cells can also be engineered to express such immune receptors in a transient way, with only a few trials evaluating the former for the treatment of ovarian cancer and malignant peritoneal mesothelioma (NCT03608618; [59]) and the latter for the treatment of colorectal cancer (NCT03415100; Table 3).

The use of mRNA electroporation for the treatment of infectious diseases has been less widespread compared to solid and hematological malignancies. DCs have been engineered with human immunodeficiency virus (HIV) antigens alone or in combination with immune-modulating molecules for the treatment of HIV infection (Table 1). Furthermore, mRNA electroporation has been used to introduce zinc finger nucleases for the disruption of CCR5, a key chemokine receptor in HIV infection, in CD4 T cells to protect the adoptively-transferred CCR5-edited CD4 T cells from HIV targeting (NCT02388594, Table 2). Only one registered clinical study relies on this technique to redirect T-cell specificity in type 1 diabetes (NCT02117518, Table 2). In preparation for clinical translation, tolerogenic DCs electroporated with mRNA-encoding myelin antigens have shown promising results in mouse models for the treatment of multiple sclerosis [60], warranting the exploration of these findings in clinical trials.

## 7. Conclusions

Electroporation of mRNA is a versatile methodology for the transient expression of proteins of interest. As a highly flexible system, it allows the fine-tuning of transfection conditions for each cell type and to multiplex mRNAs as required. The selection of the best transfection conditions for mRNA ensures maximal transfection efficiency, and thus protein expression, without compromising cell viability. As we have noted, there is a wide variety of options when it comes to improving both the electroporation conditions and stability/translation of the mRNAs for monocistronic and polycistronic gene transfer. These enhancements and different tools can be used either alone or in combination, depending on the needs of the study. Although we have focused on conventional mRNA, similar statements are true for other types of RNA, such as small interfering RNA, guide RNA in a CRISPR setting, or non-conventional self-replicating mRNA but also for purposes other than the transient gene transfer, as in gene silencing and gene disruption. The safety of the system due to its transient non-integrative approach together with its simplicity in terms of the basic equipment needed for its application ensure that mRNA electroporation will continue to be an essential method for non-viral genetic engineering in cell-based immunotherapies, especially in a clinical setting.

## Figures and Tables

**Table 1:** mRNA synthesis and electroporation conditions in clinical trials using mRNA electroporation for gene transfer in dendritic cells.

Disease	Gene(s)	mRNA Synthesis		EP Conditions		Clinical Trial Identifier and References
		Template	Production	Device	Settings	
**Solid malignancies**						
Melanoma	TAA (murine TRP2)	Linearized pING vector	mMessage mMachine T7 kit	BTX ECM 830 square wave electroporator	700 V (two pulses) 2-mm cuvette	NCT01456104 [61]
Melanoma	TAA (gp100, tyrosinase)	Linearized pGEM4Z/hgp100/A64 pGEM4Z/tyrosinase/A64 vectors	Produced by CureVac GmbH Purified by PUREmessenger^TM^ (chromatography)	Gene Pulser Xcell (Bio-Rad)	Exponential decay pulse (300 V, 150 μF) 4-mm cuvette	NCT00243529 [62]
Melanoma	TAA (h-TERT, survivin) + tumor cell mRNA	ND	T7 mMESSAGE mMACHINE large-scale transcription kit (Ambion) Purified with MEGAclear column (Ambion)	BTX ECM 830 square wave electroporator	Square wave pulse	NCT00961844 [63,64,65]
Melanoma	TAA (gp100 and tyrosinase) + immune modulating molecules (active TLR4, CD70)	Linearized pGEM4Z/hgp100/A64 pGEM4Z/tyrosinase/A64 vectors	Produced by CureVac GmbH Purified by PUREmessenger technology (chromatography)	Gene Pulser Xcell (Bio-Rad)	Exponential decay pulse (300 V, 150 μF) 4-mm cuvette	NCT01530698 NCT00940004 [62,66]
Melanoma	TAA (MAGE-A3, MAGE-C2, tyrosinase, gp100) + immune modulating molecules (CD40L, CD70, caTLR4) (TriMixDC-MEL product)	Linearized pGEM-CD40L pGEM-CD70 pGEM-caTLR4 pGEM-sig-MageA3-DCLamp pGEM-sig-MageC2-DCLamp pGEM-sig-gp100-Lamp pGEM-sig-tyrosinase-Lamp vectors	mMESSAGE mMACHINE Ultra T7 Kit Length, concentration and purity evaluated with Agilent 2100 Bioanalyzer (Agilent Technologies) using RNA 6000 Nano LabChip Kit (Agilent Technologies)	EQUIBIO Easyject Plus	300 V, 450 μF, 99 Ω (pulse time ~5 ms)	NCT01066390 [67,68] NCT01676779 [68] NCT01302496 [68,69]
Breast cancer Melanoma	TAA (hTERT, survivin, p53)	Linearized pCI/hTERT/A102 pSP73/p53/A64 pSP73/survivin/A64 vectors	mMESSAGE mMACHINE T7 Ultra kit (Life Technologies) Purified with MEGAclear kit (Ambion) Length, concentration, and purity evaluated with Agilent 2100 Bioanalyzer (Agilent Technologies) using RNA 6000 Nano LabChip Kit (Agilent Technologies)	BTX ECM 830 square wave electroporator	Square wave pulse (500 V, 2 ms) 4-mm cuvette (placed for 5 min on ice)	NCT00978913 [70]
Uveal melanoma	TAA (gp100, tyrosinase)	ND	ND	ND	ND	NCT00929019 [71]
Renal cell carcinoma	huCD40L + autologous tumor cell mRNA (AGS-003 product)	Linearized pCR2.1/CD40L wt vector from pCR2.1 (Invitrogen)	mMessage mMachine T7 Ultra kits (Ambion) Purified using RNeasy column (QIAGEN)	Bio-Rad	4-mm cuvette	NCT00272649 [72] NCT00678119 [73,74] NCT01582672 [74,75]
Renal cell carcinoma	huCD40L + autologous tumor cell mRNA (AGS-003 product)	ND	ND	ND	ND	NCT02170389 NCT01482949 NCT04203901
Bladder urothelial carcinoma	huCD40L + autologous tumor cell mRNA (AGS-003-BLD product)	ND	ND	ND	ND	NCT02944357
Non-small cell lung cancer	huCD40L + autologous tumor cell mRNA (AGS-003-LNG product)	ND	ND	ND	ND	NCT02662634
Lung cancer	TAA (MIDRIX4-LUNG product)	ND	ND	ND	ND	NCT04082182
Glioblastoma multiforme	CMV pp65-LAMP	pp65-LAMP/A64	ND	ND	ND	NCT00626483
Glioblastoma multiforme	CMV pp65-LAMP	pp65-LAMP/A64	ND	ND	ND	NCT00639639 [76,77]
Colorectal cancer	TAA (CEA)	ND	ND	Gene Pulser Xcell (Bio-Rad)	Exponential decay pulse (300 V, 150 μF) 4-mm cuvette	NCT00228189 [78]
Solid tumors (malignant pleural mesothelioma)	TAA (WT1)	Linearized pGEM/WT1 pST1/sig-WT1-DC-LAMP pST1/sig-WT1-DC-LAMP-OPT (codon-optimized version of pST1/sig-WT1-DC-LAMP) vectors	Produced by CureVac GmbH	Gene Pulser Xcell (Bio-Rad)	Exponential decay pulse (300 V, 7 ms) 4-mm cuvette	NCT01291420 [79]
Prostate cancer	TAA (PSA, PAP, survivin, hTERT)	ND	ND	ND	ND	NCT01446731 [80]
**Hematological malignancies**						
Hematological malignancies	TAA	ND	ND	ND	ND	NCT02528682
Acute myeloid leukemia	TAA	ND	ND	ND	ND	NCT01686334
Acute myeloid leukemia	TAA (WT1)	ND	Produced by CureVac GmbH	Gene Pulser Xcell (Bio-Rad)	Exponential decay pulse (300 V, 7 ms) 4-mm cuvette	NCT00834002 [81]
Acute myeloid leukemia Chronic myeloid leukemia Multiple myeloma	TAA (WT1)	Linearized pGEM/WT1 pST1/sig-WT1-DC-LAMP pST1/sig-WT1-DC-LAMP-OPT (codon-optimized version of pST1/sig-WT1-DC-LAMP) vectors	Produced by CureVac GmbH	Gene Pulser Xcell (Bio-Rad)	Exponential decay pulse (300 V, 7 ms) 4-mm cuvette	NCT00965224 [82]
Acute myeloid leukemia	TAA (hTERT-LAMP-1)	Linearized pGEM4Z/hTERT/LAMP/A64 vector	mMESSAGE mMACHINE high yield capped RNA transcription kit (Ambion) Purified with RNeasy kit (Qiagen)	Gene Pulser II (Bio-Rad)	Cells + mRNA for 5 min on ice Exponential decay pulse (300 V, 150 μF) 4-mm cuvette	NCT00510133 [83,84]
Acute myeloid leukemia	TAA (WT1 isoform A, PRAME, CMV pp65)	Codon-optimized mRNA	Produced at Oslo University Hospital	ND	ND	NCT01734304 [85,86,87]
Myelodysplastic syndromes Acute myeloid leukemia	TAA	ND	ND	ND	ND	NCT03083054
Multiple myeloma	TAA	ND	ND	ND	ND	NCT01995708
**Infectious diseases**						
HIV	HIV antigen (Gag, Nef, Vpr, Rev (GNVR)) + immune modulating molecules (hCD40L) (AGS-004 product)	HIV antigens: PCR fragments hCD40L: Linearized pCR2.1 vector	mMessage mMachine T7 Ultra kit (Life Technologies) Purified with RNeasy columns (QIAGEN)	ND	ND	NCT02042248 [88,89] NCT02707900 [90] NCT00381212 [91] NCT01069809 NCT00672191 [92]
HIV	HIV antigen (HIV-1 Gag, Nef)	Codon-optimized coding sequence including endoplasmic reticulum translocation signal peptide, antigen polypeptide, and human lysosome-associated membrane protein-1 targeting sequence	Produced by Asuragen	Gene Pulser II (Bio-Rad)	Square wave pulse (900 V, 0.75 ms)	NCT00833781 [93]
HIV	HIV antigen (Tat, Rev, Nef, Gag, NP1)	Linearized pGEM-sig-Tat-DC-LAMP pGEM-sig-Rev-DC-LAMP pGEM-sig-Nef-DC-LAMP pST1-sig-Gag-DC-LAMP pGEM-Sig-Flu-NP1-DC-LAMP vectors	mMESSAGE mMACHINE™ kit (Life Technologies)	EQUIBIO EasyjecT Plus^®^ (EQUIBIO)	12 × 10^6^ DC: 300 V, 150 μF, 99 Ω (pulse time 5–6 ms) 50 × 10^6^ DC: 300 V, 450 μF, 99 Ω 4-mm cuvette	VUB-05-001 MEC-2005-227 [94]
CMV	CMV pp65	ND	Produced by Curevac GmbH	ND	ND	EudraCT 2008-006074-15 EudraCT 2008-000430-45 [95]

Abbreviations: CEA, carcinoembryonic antigen; CD, cluster of differentiation; CMV, cytomegalovirus; DC, dendritic cell; EP, electroporation; gp100, glycoprotein 100; HIV, human immunodeficiency virus; hTERT, human telomerase reverse transcriptase; LAMP, lysosome-associated membrane protein; MAGE, melanoma-associated antigen; mRNA, messenger RNA; ND, no data; PAP, prostatic acid phosphatase; PCR, polymerase chain reaction; PRAME, preferentially expressed antigen in melanoma; PSA, prostate specific antigen; TAA, tumor-associated antigen; TLR4, toll-like receptor 4; TRP2, tyrosinase-related protein 2; WT1, Wilms’ tumor 1. Last search on clinicaltrials.gov and PubMed: 5 March 2021.

**Table 2:** mRNA synthesis and electroporation conditions in clinical trials using mRNA electroporation for gene transfer in T cells.

Condition	Gene	mRNA Synthesis		EP Conditions		Clinical Trial Identifier and References
		Template	Production	Device	Settings	
**Solid malignancies**						
Malignant peritoneal mesothelioma	CAR	Linearized pDrive vector (Qiagen) (GOI + two repeats of 3′-UTR from beta globulin (2bgUTR) with or without 150 poly(A) tail)	mMESSAGE mMACHINE T7 kit (including regular cap analog; Life Technologies) mMESSAGE mMACHINE T7 Ultra kit (including anti-reverse cap analog; Life Technologies) mScript™ RNA System (including capping enzyme and 2′-O-Methyltransferase capping enzyme to generate Cap 1 IVT RNA; Epicentre)	BTX ECM 830 square wave electroporator/Maxcyte	2-mm cuvette (BTX) /OC-400 (Maxcyte)	NCT01355965 [96,97,98]
Pancreatic ductal adenocarcinoma Breast cancer	CAR	Linearized pDrive vector (Qiagen) (GOI + two repeats of 3′-UTR from beta globulin (2bgUTR) with or without 150 poly(A) tail)	mMESSAGE mMACHINE T7 kit (including regular cap analog; Life Technologies) mMESSAGE mMACHINE T7 Ultra kit (including anti-reverse cap analog; Life Technologies) mScript™ RNA System (including capping enzyme and 2′-O-Methyltransferase capping enzyme to generate Cap 1 IVT RNA; Epicentre)	Maxcyte	ND	NCT01897415 [98,99,100] NCT01837602 [98,99,100,101]
Breast cancer	CAR	ND	ND	ND	ND	NCT03060356 [102]
Hepatocellular carcinoma	TCR	Linearized pVAX1 vector	mMESSAGE mMACHINE T7 Ultra kit (including anti-reverse cap analog; Life Technologies) Concentrated by lithium chloride precipitation Dissolved in T4 buffer (BTX)	AgilePulse Max system (BTX)	Manufacturer’s recommended protocol	NCT02719782 [103,104,105] NCT03634683 [103,104] NCT03899415 [103,104,106]
Hepatocellular carcinoma	TCR	ND	ND	ND	ND	NCT04745403
Colorectal cancer	TCR	mRNA expression vector Sequence containing 2A construct	Capping: Anti-Reverse Cap Analog (TriLink Biotechnologies Inc.)	BTX ECM 830 square wave electroporator	Square Wave pulse (500 V, 2 ms) 4-mm cuvette	NCT03431311 [107,108]
**Hematological malignancies**						
Hodgkin lymphoma	CAR	Linearized pGEM4-Z/A64 vector	mMESSAGE mMACHINE T7 Ultra kit (including anti-reverse cap analog and in vitro poly(A) tailing (“E-PAP”); Life Technologies)	Gene Pulser Xcell (BioRad)	Square wave pulse (500 V, 5 ms)	NCT02277522 NCT02624258 [109,110,111]
B-cell non-Hodgkin’s lymphoma B-cell chronic lymphocytic leukemia	CAR	ND	ND	ND	ND	NCT02315118 [112]
Acute myeloid leukemia	CAR	Linearized pDA vector	mMESSAGE mMACHINE T7 Ultra kit (including anti-reverse cap analog; Life Technologies) mRNA purified by RNeasy Mini Kit (Qiagen)	BTX ECM 830 square wave electroporator	2-mm cuvette	NCT02623582 [113,114,115]
Multiple myeloma	CAR	Linearized DNA plasmid Codon-optimized nucleotide sequence containing 3′-UTR, mouse alpha globin 5′-UTR, and poly(A) tail	ND	ND	ND	NCT03448978 [116,117]
**Autoimmune diseases**						
Type 1 diabetes	Peptide-MHC-CD3-zeta construct	ND	ND	ND	ND	NCT02117518
**Infectious diseases**						
HIV	ZFN	Linearized pDA-A.2bg.150A vector	mMESSAGE mMACHINE T7 Ultra kit (including anti-reverse cap analog; Life Technologies) mRNA purified by RNeasy Maxi kit (Qiagen)	MaxCyte GTTM Flow Transfection System	ND	NCT02388594 [118]

Abbreviations: CAR, chimeric antigen receptor; CD3, cluster of differentiation 3; EP, electroporation; GOI, gene of interest; IVT, in vitro transcribed; MHC, major histocompatibility complex; mRNA, messenger RNA; ND, no data; TCR, T-cell receptor; UTR, untranslated region; ZFN, zinc finger nuclease. Last search on clinicaltrials.gov and PubMed: 5 March 2021.

**Table 3:** mRNA synthesis and electroporation conditions in clinical trials using mRNA electroporation for gene transfer in natural killer cells.

Condition	Gene	mRNA Synthesis		EP Conditions		Clinical Trial Identifier and Reference
		Template	Production	Device	Settings	
Colorectal cancer	CAR	PCR product from pFBCMV-T7 vector GOI + 5′-UTR with Kozak sequence, and ClaI, the GM-CSF signal peptide encoding sequence (SP) and the alpha-globin 3′-UTR sequence	mMESSAGE mMACHINE T7 Ultra kit (including anti-reverse cap analog (ARCA); Life Technologies) mScript™ RNA system (Epicentre)	NEPA21 electroporator (Nepagene) BTX electroporator (AgilePulse)	2 or 4-mm cuvette	NCT03415100 [119]

Abbreviations: CAR, chimeric antigen receptor; EP, electroporation; GOI, gene of interest; mRNA, messenger RNA; ND, no data; UTR, untranslated region. Last search on clinicaltrials.gov and PubMed: 5 March 2021.

## Data Availability

Data sharing not applicable.
